# Ethics, health policy-making and the economic crisis: a qualitative interview study with European policy-makers

**DOI:** 10.1186/s12939-019-1050-y

**Published:** 2019-09-14

**Authors:** Caroline Brall, Peter Schröder-Bäck, Rouven Porz, Farhang Tahzib, Helmut Brand

**Affiliations:** 1 Department of Health and Technology, Health Ethics and Policy Lab, ETH Zurich, Hottingerstrasse 10, 8092 Zurich, Switzerland; 2 Department of International Health, CAPHRI – Care and Public Health Research Institute, Maastricht University, Maastricht, The Netherlands; 3 Faculty for Human and Health Sciences, University of Bremen, Bremen, Germany; 4 Clinical Ethics Unit, Bern University Hospital Inselspital, Inselgruppe AG, Bern, Switzerland; 5 UK Faculty for Public Health, London, UK

**Keywords:** Policy-makers, Ethics, Austerity, Economic crisis, Decision-making

## Abstract

**Background:**

The economic crisis posed various challenges to policy-makers who had to decide on which health policy measures to focus on and on which to refrain from. The aim of this research was to assess the relevance of ethics and to highlight ethical dimensions in decision-taking by policy-makers with regard to policy and priority-setting in health systems posed by the economic crisis.

**Methods:**

Semi-structured qualitative interviews were conducted with eight European policy-makers from six countries.

**Results:**

All interviewees recalled difficult and strenuous situations where they had to prioritise between distinct areas to focus on and invest in, for example around choices between prioritising medications, health professional staffing, care specific equipment, or urgent infrastructure issues. Values could be identified which they deemed as important within the policy-making process, such as trust and responsibility. They explicitly expressed the need for ethical tools and assistance in terms of policy advice for reaching morally sustainable decisions in health policy matters.

**Conclusions:**

The study showed that ethical concepts and values frequently come into play in health policy-making, and that ethics is highly relevant in policy-makers’ daily decision-taking, yet that they lack ethical guidance on what to base their decisions. The study is of relevance since it can provide future decisions on austerity-related issues with an ethical underpinning and could identify areas of moral concern.

## Background

Countries in Europe have employed different policy responses to the economic crisis that set off in 2008. Whereas Iceland or Sweden, for example, chose financial stimulus and consequently invested in social safety nets etc., countries including Portugal, Spain, Ireland and Greece had to employ austerity measures as part of structural rearrangements so as to receive funds issued by the International Monetary Fund (IMF) [1, 2]. For the latter countries, the economic policy as a response to the crisis was to lower spending in social welfare areas, for example as in the case of Greece where many cutback measures of health programmes, such as HIV programmes, were implemented [3–5].

Such challenging health system modifications were shaped by policy-makers, who decided what measures and approaches to implement in order to meet their often restricted budgets for health. One can assume that public policy-makers with designated responsibilities for health policy had to confront various moral dilemmas in their decision-making. Especially in times of austerity, with its increase of inequalities and questions of just allocation of resources, the relevance of ethics in policy-making is predominant even more. As Sullivan and Segers describe, politicians and policy-makers act in a “distinctive realm” as they “act for others but also serve themselves, they rule over others and can coerce people, and their decisions have broad, cumulative effects on present and future citizens” [6]. Previous research has shown that they often feel largely unprepared to reflect their decisions ethically [7].

In this context, the aim of this research was to assess the relevance of ethics and to highlight ethical implications in policy-makers’ daily conduct with regard to the challenges in terms of policy and priority-setting in health systems posed by the economic crisis. Ethics here is understood as the discipline that critically and systematically reflects on the morals of people. Morals are the values, norms, principles and ideals that a person holds. Ethics thus aims at spelling out “standards of good and bad, right and wrong. Normative ethics tries to offer a substantive, albeit general answer to the question, What should I do?” (Jennings, 2003).

Apart from the European Union (EU) values for health, namely solidarity, universality, equity, access to good quality care [8], there is no specific explicit set of moral values or professional moral value system established for policy-makers – besides their individual or party’s perception of the good and a political party affiliation for some. Thus, the aim of this study is to descriptively explore what the principles, rules, ideals and values of health policy-makers are and what role they play in the decision-making during times of austerity in Europe. In view of the lack of research about policy-makers’ moral values, it is necessary to uncover the relevance of such values in policy-making, assessed by means of the pressing example of the economic crisis and its impact on health and health care systems in Europe.

## Methods

In order to pursue the study’s aim a qualitative, explorative approach was selected. An interview study with EU policy-makers aimed to assess how they a) perceived the economic crisis with regard to health, b) which values they perceive as essential to be integrated in policy-making during challenging situations with regard to priority-setting and decision-making in health policy matters, and c) how they evaluate the role of ethics in political decision-making.

### Data collection

Accordingly, semi-structured telephone or video-supported telephone interviews with policy-makers at European, national or regional level within EU Member States were conducted. As policy-makers, persons were labelled who currently work or have worked in high-ranking positions related to health policy at a governmental institution.

The interviewees were located according to two sampling methods. Firstly, a purposively sampling approach of maximum variation was adopted based on the participant’s function and country of origin (recipient/donor country, austerity implemented or not). In this first stage only current or former Members of the European Parliament with expertise in health policy were contacted, since the initial aim was to focus on their perceptions of the crisis on the European Union level exclusively. During this stage, 80 potential participants were individually contacted by email including a cover letter and a reminder email 2 weeks afterwards. Emails were sent out during two phases in mid 2015 and early 2016. While 9 emails could not be delivered due to expired email addresses, the majority did not respond or declined the invitation due to lack of time. After having achieved a response rate of 3.8% (*n* = 3), a snowball sampling technique was embraced in a second approach for accessing possible interview partners. Additionally, the sample was broadened from involving former or current Members of the European Parliament only to including other health policy- or decision-makers who work at European, national or regional level in the European region. By this, sufficient interviews could be collected to reach thematic saturation (see results).

For conducting the interviews, a semi-structured interview guide was developed, which was clustered into three broad themes, precisely (1) values, including EU values for health, (2) ethical decision-making and priority-setting during the economic crisis with regard to health, and (3) an evaluation on the usefulness of ethics assistance. According to Flick, a semi-structured interview can be seen as a way to iteratively reconstruct ‘subjective theories’ about a set of themes, which is in line with the goal of this study [9]. Data was sought to be retrieved about how European policy-makers perceive the situation of austerity measures in health care, to reveal whether ethics plays a role therein in their view, and in how far ethics could help in taking decisions in this regard. In order to ensure reliability, the interviews were recorded, transcribed and stored anonymously at a secured place.

### Data analysis

The collected information was summarized and categorised according to emerging categories and subcategories. By applying a content analysis in line with Mayring, policy-makers’ perceptions about the questions outlined above could be revealed [10]. For this, all interviews were read as a whole to gain an initial impression of the concepts in a first step. In a second step, categories and subcategories were coded in each interview. After a thorough re-analysis, the emerging categories and subcategories were discussed and validated by four researchers (CB, PSB, RP and HB).

### Ethical considerations

The study was conducted in compliance with the Declaration of Helsinki and was reviewed by the medical ethics committee of the University Hospital Maastricht and Maastricht University (METC azM/UM) on December 19th 2014 (METC 14–5-097). In the invitation letter study participants were informed about the aims and procedures of the study, were given the choice whether to participate or not and were ensured about their anonymity. Their verbal consent was obtained in the beginning of the interviews and was recorded accordingly. (No written consent form could be obtained for all interviews due to the fact that interviews were held via telephone where printing, signing and scanning consent form would have been additional burden for participants).

## Results

In total, 8 semi-structured explorative interviews were conducted between February 2015 and September 2016 until thematic saturation was reached, meaning that a sufficient number of persons were interviewed so that important features of the investigated topic could be revealed. According to Guest et al. thematic saturation in explorative interviews appears between six and maximum 12 interviews [11]. The length of the interviews varied between 28 min and 1 h 18 min. Participants from 6 different countries could be sampled, covering several geographic regions within Europe: West (Great Britain, The Netherlands), South (Italy, Portugal, Malta), Central/East (Slovenia). Participants moreover show a mix of functions and level of actions, including (former) Members of the European Parliament, policy advisors or civil servants working at European, national and regional levels in health policy related matters. Table 1 presents an overview of the study participants, including their highest attained function during their career to date and level of action. Country and gender were purposively not declared explicitly in the overview so as to increase anonymity of participants.

**Table 1:** Overview of study participants

Participant	Function	Level of action
P01	MEP	EU & national
P02	MEP	EU
P03	MEP, Government advisor	EU & national
P04	Civil servant	National
P05	Civil servant	National
P06	Civil servant	EU
P07	Civil servant	Regional
P08	Member of government	EU & national

In the conducted interviews, six themes emerged from the data which were most significant, namely: 1) examples of difficult decisions in policy-making brought about by austerity measures, 2) decision-making and priority-setting, 3) ethics in dealing with decisions, 4) values in policy-making, 5) EU values for health, 6) recommended health policy measures in times of crisis. Table 2 gives an overview of the identified categories and subcategories, which will be described more thoroughly in the following.

**Table 2:** Summary of categories and sub-categories

Category	Sub-category
Examples of difficult decisions in policy-making brought about by austerity measures	- Medication - Recruitment/staff - Health care provision sites
Decision-making and priority-setting	- Perception of decision-making and priority-setting as challenging - Process of priority-setting - Power plays between different stakeholders - Lobbying
Ethics in dealing with decisions	- Importance - Need for ethics assistance
Values in policy-making	- Political party’s vs. individual values - Lack of value set - Economic vs. ethical values
EU values for health	- EU values as passively guiding action - Solidarity and responsibility
Recommended health policy measures in times of crisis	- Recommendations for specific health policy measures, overall spending and other health level increasing measures

All interviewees^1^ reported an exceptionally difficult time for policy-makers with regard to having to make tough decisions in the aftermath of the economic crisis since 2008. They reported that the most challenging time for them was in the years 2011 and 2012, when budgets were restricted:

*“There was a real cash problem 2011/2012. It was not immediately after the crisis, but it was the time when we were in the exit of the procedure and budgets were basically restricted.”* (P05)

### Examples of difficult decisions in policy-making brought about by austerity measures

Giving examples of those tough decisions they had to take, they differentiated between making trade-offs in the areas of medications, health care staff and quality of health care provision. As one interviewee reported, prioritisation had to take place with regard to the medicines bought by government. The interviewee P05 perceived those decisions as prioritising between life and death.

*“There was a time when there was no budget to buy all the medicines. And every week I had to give directions on which medicines to prioritise. And in such a situation I can give a concrete example, I would say: Don’t buy the statins, don’t buy the anti-hypertensives. Those people can afford to buy and if they keep their statin for a month or two, it’s not the end of the world. But I can’t afford not to have medicines in my ICU [intensive care unit], in my special care baby unit, in my ED [emergency department]. That’s the kind of example. Yes, unfortunately for a few months, I was in that very, very difficult situation. You have to prioritise what is a matter of life and death.”* (P05)

The interviewee pointed out another area where prioritisation was a challenge: recruitment of personnel:

*“What do you recruit? And again, the priority was always to recruit the doctors and the nurses. We would leave the physiotherapists, the dieticians, we would leave those for a later time. The first money available was always going first for the doctors and the nurses because that was key impact.”* (P05)

Another interviewee stressed that it was challenging to still provide a sufficient level of quality in health care provision, which had to be at the cost of other health care provision sites:

*“Main challenges [with a reduced budget] are drug prices, staff prices, because we don’t have money to have more staff, medical equipment, the old medical equipment cannot be repaired or be changed, and obviously at the end of the day quality of services - they will be affected. And again for very good hospitals the top level of the quality, it’s difficult to protect. So usually the idea is to cut small hospitals and to invest more into big hospitals. But then […] citizens are complaining, 'Oh do I have to take a car and drive 20, 30, 40 km, why do I have to do so?'. I close a small hospital and I invest more money in a big hospital, difficult.” (P07)*

Personally, they perceived that time as challenging with responsibility on their shoulders, as one interviewee noted the importance of making the choices himself, rather than leaving it up to colleagues.

*“That was a horrible time. I still look back and wonder how I coped. But the choice I had at that time was either to make the decision myself, or to leave it in the hands of people who would make it randomly.” (P05)*

### Decision-making and priority-setting

When asked about their perception about decision-making and priority-setting, most policy-makers considered it as very tough, even referring to it as a ‘nightmare’ when making decisions for or against certain types of population. Making decisions was however seen as inevitable.

*“I mean, if you like, it’s a nightmare – decision-making. Because there always will be cases like that where you have to weigh one lot of vulnerable people against another. And that’s neither easy nor pleasant and if you are in government or running a health service, you have to take the decisions. [...] You do have to take decisions. Otherwise the system breaks down.” (P01)*

A theme raised by interviewees when speaking about priority-setting was the importance of the process. Here, an important trait within the process was identified as ‘listening’ to others, e.g. patients, colleagues, professionals etc. in order to attain information and be able to negotiate in a second step.

*“So you listen a lot, you listen to the patient but you also listen to the health professionals, you listen to the managers, you listen to the specialists, you listen to the academics, and you try and listen. Your job as a politician is to listen and learn and then ultimately to make decisions based on what you’ve heard and what you know and then what you can negotiate with other people in government. It starts with listening and ends with negotiating.” (P01)*

On the other hand, one interviewee stated that listening to others results in difficulties for policy-makers to hold on and continue acting according to what they themselves perceive as right, but rather are driven externally by a power play between citizen interests, media, associations and more and their respective interests.

*“I think that most politicians, at least well prepared politicians, they know what the priorities are, they know how it should be done. And when they are in the opposition, they always know in a very clear way. But when they become government and they have to take the decision on their own and they have to be responsible for the decision, they always look around. And to look around is, once again, to media and public opinion. So instead of going the way they know they have to go, they go a little bit to the side of what is popular in media and in public opinion once again. […] So, this [is] a game of power. Political power, citizen’s power, associations, lobbies, economic interests. This is a game of power.” (P03)*

The majority of policy-makers also mentioned lobbying as a central element during the priority-setting process. In their comments, they implicitly referred to the criteria of trust, transparency and legitimacy as important with regard to lobbying. Another sub-theme raised with regard to priority-setting criteria was how decisions are reached and the role of procedural values to guide this process. While some policy-makers deemed procedural values as important, others thought they are rather needless. The procedural value granted most importance by the interviewed policy-makers was accountability. All in all, they express the need for objective criteria to base decisions on during priority-setting.

### Ethics in dealing with decisions

The importance of ethics in dealing with difficult decisions during policy-making, not only during times of crisis but during political decisions in general, was perceived by all interviewees as high. What is more, they consider ethics as a helpful tool to guide decisions in making trade-offs, which cannot be prevented when money is not available for all needed areas. That deciding in which areas to invest and ethics are closely interlinked – that there is even an ethical nature inherent to such decisions – is a theme which emerged during all interviews as the selected following quotes depict.

One interviewee described it as an ethical responsibility to balance between areas to invest in:

*“Investments in health have to be balanced with all the other societal needs. And that is a responsibility of ethics.” (P02)*

Another interviewee also referred to balancing of investments as making trade-offs.

*“Trade-offs are part of ethical behaviour.” (P06)*

Moreover, one interviewee referred to the importance of ethical decision-making especially in times of economic scarcity, with specific emphasis on upholding equity.

*“I think in times of austerity the ethics of decision-making becomes even more important. Because very often one is having to make difficult decisions between spending areas or projects and so it’s important when one is making most decisions one takes into account what is equitable.” (P01)*

When asked about whether ethics could be helpful in their decision-making, they expressed the need for specific advice, support and assistance in considering ethical dimensions. Regarding the form of such ethical assistance, retreats, master classes and workshops were mentioned as favourable and helpful in discussing and analysing involved values and potential options of decisions to be taken. Such assistance can help to educate and train policy-makers in identifying and prioritising values and norms during their decision-making. One interviewee suggested that by receiving ethics assistance he would feel less ‘troubled’ during the decision-making process. The interviewee suggested that by having better knowledge about the underlying ethical concepts he would gain more confidence in making decisions.

*“I think had I before gone through the process, being exposed for example to some workshop or something for senior policy-makers, which would have introduced the concept that in times of crisis different value sets may need to come into play, that it's okay to depart from the established norms and to work in a different reality, maybe I would have felt personally less bad and less troubled and would have been more able to cope on a personal level with the decisions that I would having to make. […] These kind of master classes, retreats if you wish, for senior decision-makers involved in making these very tough decisions, could be a very useful kind of support.” (P05)*

### Values in policy-making

Reflecting on their policy- and decision-making during times of austerity, all of them reflected on the values considered at the time. The degree to which this was done however is diverging, ranging from very explicit reflection and mentioning of values to rather implicitly revealing them.

Some policy-makers – the ones following or being involved in a political party – reported that they make distinctions between their political party’s values and ideology and their individual values. One interviewee stressed the fact that when their personal moral values conflict and clash with their political party’s values, this would be often at the expense of personal values.

“*When talking about the difference between a personal ethical decision and the party’s ethical decision, you try and argue within your party of course for the ethical decision, but if the majority goes against you, sometimes you have to say ‘Okay I lost that argument, so I go along with the majority for the moment, then I raise it again later’. So it’s sometimes that you put your personal view of the ethical decision on ice but without residing from it completely.” (P01)*

While it is nothing new that often political party’s values take precedence over individual values, such fact has however not been empirically confirmed and helps to better point towards moral conflicts in policy-making processes and their underlying causes. One interviewee regarded the value systems in place as disappointing, as values can be rejected due to strategic considerations to reach political or personal goals.

*“For me it was quite a disappointment to see that we don’t really find a cluster of values that are always present in the politician decision. Although each one has their own values – mostly more political values than moral values – politicians in general do not really make a sharp distinction between moral values and political values. So although they have them, they can just be bent over when other interests are in place. And those interests can be national interests, and also ideological interests, and also the way they believe that their decisions might be perceived by the public in their own countries.” (P03)*

Another interviewee also stressed the constant dilemma between values and principles, both for the individual policy-maker him- or herself, i.e. to balance between different values which are important to him or her, and at the collective level when negotiating with other stakeholders.

*“So at the highest level of decision-making and policy-making it is a permanent battle of interests, it’s a permanent dilemma between values, principles, promises and so on. And that of course has everything to do with politics, setting priorities, which is making decisions, but the implications could hurt enormously parts of the population. So what is justice, what is real, what is honest, appropriate? That is a permanent ethical battle at an individual level of the policy-responsible such as a minister, but also a collective one, such as the health care insurance or the governments. There is no politics without ethical stance or positions you could take and ethical principles.” (P04)*

Relating to political decision-making, the issue of trust was raised by several interviewees, precisely the importance of trust when it comes to arrangements between colleagues and maintaining trust with regard to confidentiality, as stated by interviewee P06.

*“And there, it’s almost like a confession in church, you don’t go outside and say ‘Oh Mrs. X says she needs help in this area’, you might say that to the people who could give her the help, but you won’t say that just publicly because in a way there is a degree of confidentiality between you and your constituents. You want to maintain that level of trust. So all these things, they are straightforward in theory, but are not so straightforward in practice.” (P06)*

On the other hand, trust was regarded as a central element at the policy level at large. As another interviewee stated, trust is closely related to having and acting according to a certain set of values. A lack of trust was even depicted as as a major failure in politics. Acting to a certain set of values increases trust for policy-makers, while trust is vice versa a necessary prerequisite for valuable policy-making.

*“For myself, the most important thing is to have a core set of values to which we are faithful, that we publicize, so we tell voters what are our values. And we are coherent in all our decisions, so we stick to those values. This is the only way to raise trust between the politicians and the common citizen. And without trust there is nothing valuable in politics, at least from my point of view. […] Trust is the main failure in politics for me.” (P03)*

As regards different kinds of value sets that are applied in decision-making, interviewees reported that economic values are more in the foreground than social or ethical values.

*“Economic values take the immediate priority.” (P05)*

They however regarded ethics as important in dealing with difficult decisions. They particularly noted that the use of a set of criteria derived from ethics could be helpful.

*“Ethics should be a very important aspect of politics because ethics is a set of criteria that could allow politics to take correct and balanced decision in terms of investment. Profit is taken most of the times as the only criterion. We should find some mitigation of this absolute criterion of profit.”* (P02)

The interviewee’s perception was that during the aftermath of the economic crisis and its resulting austerity measures investments in physical assets and orientation towards GDP are overestimated. Instead, he proposed to invest in ‘immaterial’ goods relevant to society. He saw this as beneficial for society as a whole, as it ensures that each individual receives support to keep up its potential and thus be able to contribute to society.

*“We have to train our parameters for investment in the sense that we until now have been oriented by the GDP, which is based on the physical output. […] So we somehow have to change our criteria for investment not only to be in the physical investments, but also in investment into immaterial investment. That is mainly investment on the person, taking into consideration the dignity of the person and other immaterial goods that are in the domain of common good and commons. That is something that also reflects on the investments in the national health services because that is a means to invest in the person to keep the person to its highest level of contribution to society.” (P02)*

### EU values for health

Besides the values to employ, the interviewees also stated their perception of the EU values for health. Half of the interviewees deemed them as helpful in implicitly guiding action, whereas the other half perceived them as a lip service only, as one interviewee put it:

*“I do have some doubts about the way they are used, just like slogans, just to be in the right jargon.” (P03)*

Interviewee P08 proposed to change the overall guiding values in European health systems from a more economic oriented system towards one focussing more on the patient and accountability:

*“They [the EU values] should be important, but nowadays in the real world they are not important. Being honest, we are struggling to include the patient in the health care system. […] It might be that we should rethink the main values in the health care system. It should be moving from efficiency and productivity to quality, safety, transparency, accountability, fairness, and other things should be added.” (P08)*

The value of solidarity was discussed more in depth in terms of its application towards health during the economic crisis. Here, it was regarded as not sufficient in dealing with health on an organisational macro level. The value of responsibility was considered as more important when it comes to negotiations between different countries on EU level.

*“There are two values [solidarity and responsibility] that for me are fundamental and they ground the development of the European Union. Solidarity, because we want to become one. 28 member states that should function as one. […] And we cannot really expect just to put our hands and beg for something, without giving something else with the other hand. So if we want to expect solidarity from others, we do have to show that we are responsible in the decision, in our decision-making. We are not just asking and spending the way we just feel it’s right. […] Now it’s your turn to prove that you are responsible and that you achieve those goals in the period of time that was given to you. This is responsible behaviour. But if you and me, we just decide something together, and then - without telling you anything - I just go the other way, how do you feel? Between states it’s the same thing.” (P03)*

### Recommended health policy measures in times of crisis

The last theme embarked upon by the interviewees was what they perceived as ‘good’ measures in terms of health policy as a response to the crisis. Among those recommended health policy measures were 1) the prioritization of vulnerable groups, 2) health literacy and empowerment as an instrument for saving costs, 3) and ensuring a minimum level of health care which is accessible for anyone. The latter includes safeguarding the provision of basic rights in health care. Moreover, it was recommended to 4) increase the overall spending on health, while also changing criteria for enabling more investments towards health services. In terms of overall spending, 5) expenditure in other sectors, e.g. defence, should be reduced, however, 6) waste in health spending should be reduced at the same time. Lastly, 7) no cuts should be made in preventive and primary care, as it only results in more ill cases in the end, and 8) the importance of public opinion and the often negative power of media has to be taken into account so as to maintain or achieve appropriate support for policy measures undertaken.

## Discussion

The research presented above is – to our knowledge – the first interview study involving policy-makers and assessing their perceptions of decision-making regarding health and ethics during economic crisis. Previous studies have assessed the perspectives of health care professionals on austerity measures in health care provision [12, 13], with one study shedding light on professionalism and ethical issues encountered by health care professionals [14]. None has, however, analysed policy-makers’ perceptions of their conduct regarding health policy-making in times of austerity in ethical terms.

The data derived in this qualitative interview study shows that ethical concepts and values frequently come into play in health policy-making and ethics is thus highly relevant in policy-makers’ daily conduct, particularly in times of scarce economic resources. They perceived the consequences of the economic crisis as limiting health levels of the population and restraining health care provision in general. All interviewees recalled difficult and strenuous situations, where they had to prioritise between distinct areas to focus on and invest in, e.g. medications, health professional staffing or care equipment and sites. Their approach to how to deal with scarce resources depends on the underlying ideology of the respective policy-maker, political party’s or the country’s policy system behind: Political outcomes depend on which ideologies and concepts of justice are employed. Aiming to align those ideologies on individual, party or country level would not be suitable and desirable, however ethics could help to analyse and hence better understand the respective concepts of justice and value systems in place. When evaluating the policy recommendations proposed in the interviews, one can see that policy-makers involved in this study rather act and argue in line with concepts reasoning for a social minimum (for instance in line with utilitarian or egalitarian liberal theories).

In general, the interviewees rather refrained from explicitly mentioning *what* they decided or according to which concept of justice, but instead focused more on *how* decisions were reached. We would have expected that policy-makers talked more about their own difficult decisions taken and for which outcome (the *what*) they had decided. That they rather refrained from expressing the outcomes of their decisions could potentially be explained by the fact that policy measures and their outcomes differ from individual to individual taking the decision and based on their respective ideological understanding. Potentially, they knowingly did not want to put forward their own ideological understanding in such a study assessing general concepts of ethics in policy- and decision-making. The recommendations given for health policy measures during economic crisis are however coloured by their ideological perception. In this respect, it could generally be observed that the interviewees did not submit to ‘hard’ austerity measures in line with neoliberal ideologies, which emphasize the role of free markets and less government support. They rather promoted policies that provide a social minimum (as mentioned above). The interviews moreover showed that interviewees actively asked themselves ‘what should I do’, which mirrors what we defined by ethics. They explicit reflect on norms and values involved in their decision-making and also reasoned about methods typically practiced in ethics, such as balancing between values and reasoning about possible trade-offs. This engagement with ethical practices in policy-makers’ critical reflection confirms the overlap of policy-making practices and philosophical ethics.

As regards *how* decisions are taken within the policy process, the interviewees could take a more general view, where they altogether mentioned values or general conditions, which they perceive as essential (to the policy-making process). Trust, and thus accountability, between involved stakeholders are perceived as integral. Trust was understood as confidentiality between stakeholders when discussing topics, as well as doing what one said one would do. Strengthening trust and accountability values could be a first step to provide the ethical base for decision-making processes. A balance between solidarity on the one hand and responsibility on the other was also considered important, as they go hand in hand according to one interviewee. Responsibility has to be shown by policy-makers, political parties or EU member states complying with the decisions taken.

With regard to setting priorities within the decision-making process, resources were allocated according to what policy-makers deemed as having ‘key impact’. We interpret their definition of key impact as measures which maximise population health, e.g. as investing in doctors and nurses, who could offer treatment of the health condition, instead of physiotherapists, who usually offer supporting services for improving treatment and healing. Hence, they act according to a utilitarian, egalitarian approach to setting priorities. Apart from the policy-makers own perceptions of how to make decisions, they however also take into account the power plays both between certain stakeholders within their own party as well as within society in general, such as media, voters, associations, lobbying parties among many more. Their own perception of the ‘best’ decision might then be placed in the background. Moreover, they are disappointed by the fact that economic values are usually more important than social values.

According to the interviewed policy-makers, the need for ethics assistance in terms of tools or advice is increasing. They deem the degree of objectivity provided by ethical analysis as facilitating their decision-making tasks. In psychological terms, this could possibly be explained by objectivity giving them an increase in confidence when being in charge of tough and often emotional decisions. Regarding the form of such ethical assistance, retreats, master classes and workshops were mentioned as favourable and helpful in discussing and analysing involved values and potential options of decisions to be taken.

Despite the valuable information yielded in this interview study, some limitations should be mentioned. A first obstacle was the data collection itself. Only few interviews could be obtained, which might be due to the fact that scientific interview studies are rarely done with policy-makers. Policy-makers were often not available for research, giving the reason that they have time constraints. In view of the fact that closer collaboration between researchers and policy-makers is needed [15], the availability of policy-makers for research should be emphasized in the future. Previous studies have noted that such collaboration is difficult to achieve in practice, as objectives differ and distinct languages and frames of reference are used by policy-makers and scientists [15]. The discipline of (applied) ethics could help here as a bridge building instrument between science and policy-making, as it stems from the research arena but tries to address real-life discourses. Public health ethics frameworks can be used to help policy-makers to address those ethical issues in real-life decision-making [16]. Other methodological limitations concern the study sample. Political talking behaviours should be regarded as a constraining factor for valid information, as well as the self-selection bias of participants during the sampling process, implying that those who have an interest in ethics might have been more likely to participate in the study. During the interviews, a certain degree of social desirability bias occurred, which nearly always is the case with regard to questions involving sensitive information. By guaranteeing anonymity to the interviewees, it was tried to minimize the degree of socially desirable answers. Given the small number of interviewees, the sample is not representative and results not generalizable, yet the derived qualitative data give a sufficient level of insight into the research questions posed.

Despite those limitations, it should be noted that the study is the first empirical and qualitative assessment of ethical concepts within health policy-making during economic crisis and therefore adds an important piece to the current state of research. Future directions for research could orientate towards ethical assessments of specific high-level decision-making processes or a larger study assessing policy-makers' conduct and behaviour in policy-making processes, involving a greater number of participants. In the practice field, it would be valuable to integrate approaches of ethical support in diverse policy-making processes.

## Conclusion

Policy-makers taking decisions in public health or health care, feel that they have to decide on ethical issues permanently, especially with regard to issues concerning resource allocation in times of poor economic resources due to crisis and austerity.

Values could be identified, which they deem as important within the policy-making process, such as trust and responsibility. Policy-makers explicitly express the need for ethical tools and assistance in terms of policy advice for reaching morally sustainable decisions in health policy matters.

The study is of relevance, since it can provide future political decisions on austerity-related issues with an ethical underpinning and could identify areas of concern, which might be at the expense of maintaining or achieving health.

## Data Availability

The transcribed interviews are not publicly available, since no consent was given for that purpose.

## Footnotes

1 In order to increase anonymity, only the male form “he” will be used for all interviewees (whether male or female) in the following.
